# My research experience in China’s Space Station

**DOI:** 10.1093/nsr/nwag575

**Published:** 2026-09-07

**Authors:** Hongzhang Zhang

**Affiliations:** Hongzhang Zhang is a professor at the Dalian Institute of Chemical Physics, Chinese Academy of Sciences, and a payload specialist of the Shenzhou-21 crew

## Abstract

Hongzhang Zhao recounted his six-month experience aboard the China Space Station as a payload specialist.

On 31 October 2025, together with Lu Zhang (张陆) and Fei Wu (武飞), I boarded the Shenzhou-21 spacecraft and headed to the China Space Station (CSS). This mission was the 6th manned flight in the application and development phase of the CSS, and the 37th flight mission of China’s manned space program.

**Figure 1:**
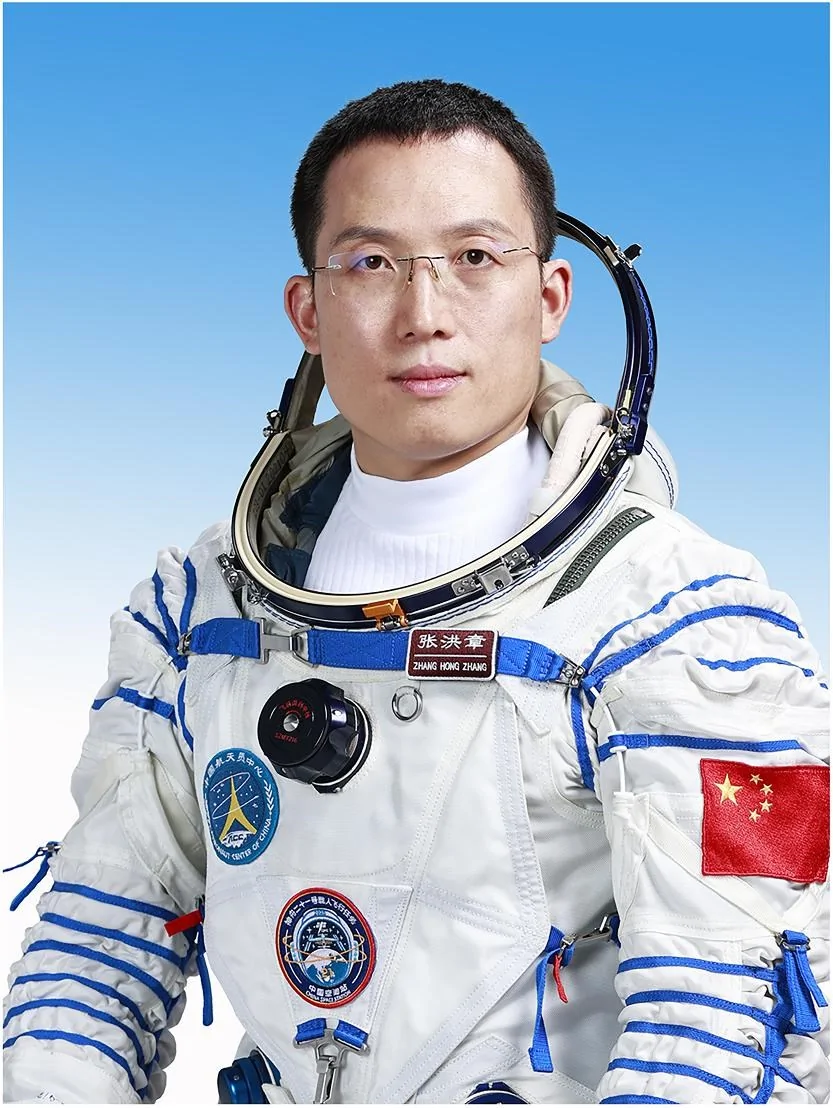
Hongzhang Zhang is a researcher in the field of high-performance batteries, and a payload specialist of the Shenzhou-21 crew.

The primary objectives of the mission were: to conduct an in-orbit crew rotation with the Shenzhou-20 crew; to reside aboard the CSS for ∼6 months; to carry out space science and application research; to perform crew extravehicular activities (EVAs) and cargo transfer operations; to install space debris protection devices, as well as install and retrieve extravehicular payloads and equipment; to conduct science popularization, education, and public welfare activities; and to carry out space-embedded experiments—all aimed at continuously maximizing the comprehensive application benefits of the CSS.

As a payload specialist, my primary responsibility was to complete the in-orbit experimental tasks, which would not have been possible without the close collaboration of my two crewmates and the support from ground-based research teams. We carried out 27 planned science and application projects, conducting in-depth and systematic research on key scientific issues across multiple fields, including space medicine, space life sciences and biotechnology, space materials science, microgravity fluid physics and combustion science, and new aerospace technologies.

Throughout the entire mission, I came to realize that the Space Station differs greatly from a ground-based laboratory, and we faced numerous challenges while conducting research in space.

The first challenge was that space-based experimental projects have extremely stringent safety requirements. The experimental equipment and samples must be able to withstand the mechanical shocks generated during the launch and reentry process of the spacecraft, and must not produce any substances that could threaten the safety of the spacecraft or the space station.

Taking lithium-ion battery research—my own research field—as an example, it is essential not only to prevent the batteries from smoking or catching fire, but also to avoid the release of harmful gases that could cause inhalation injuries to the astronauts. We have established reliable contingency plans for all potential problems.

The second challenge was that the cost of space-based experiments is extremely high. It includes not only the enormous space–ground transportation costs but also the expensive onboard energy and communication bandwidth, as well as astronauts’ working hours.

To control these costs, the experimental equipment was designed to be integrated, miniaturized and automated to reduce the size and weight of the devices and minimize the time demands on astronauts. At the same time, thorough ground-based experiments must be conducted to eliminate potential failures as much as possible.

Third, microgravity fluid effects make experiments more difficult. In a microgravity environment, many physical and chemical processes behave in fundamentally different ways. Some phenomena disappear, such as density-driven convection, thermal convection, and buoyancy; while some others become prominent, including intermolecular interactions such as surface tension and interfacial adsorption.

**Figure 2:**
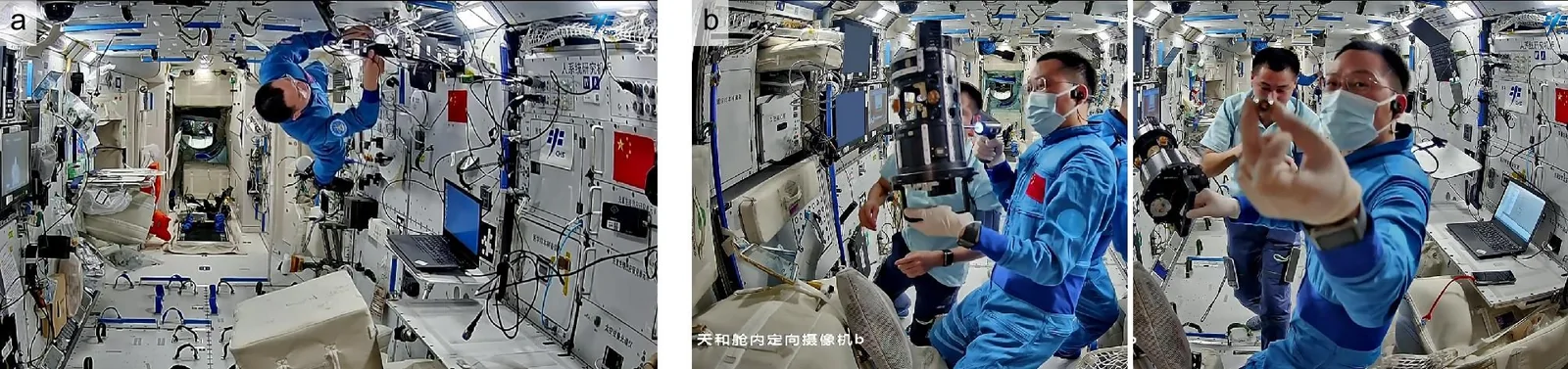
Hongzhang Zhang conducting experiments in the CSS: (a) *in situ* electrochemical experiments on lithium-ion batteries; (b) experiments in the containerless experiment chamber.

These phenomena can pose significant difficulties for space experiments and must be avoided through experimental design. For example, on the ground, it is simple to drip a drop of liquid from a straw, but aboard the space station, that drop may cling to the wall of the straw and be difficult to detach; on the ground, air bubbles in water will automatically rise to the surface, whereas in the space station, water–gas separation often requires centrifugation.

The fourth challenge relates to changes in heat transfer mechanisms. Natural convection disappears in a microgravity environment, so heat-generating materials can only dissipate heat through thermal conduction and thermal radiation. The low heat dissipation efficiency makes it easy to form localized hot spots in materials. Such hot spots may lead to problems such as runaway reactions, side reactions, or failure of electronic components. Therefore, active thermal control systems (such as forced convection, liquid cooling loops, and thermoelectric coolers) are often required to maintain stable reaction temperatures.

The fifth challenge comes from the changes in mass transport and reaction kinetics. Due to the absence of natural convection, the replenishment of reactants and the removal of products rely primarily on slow molecular diffusion, making reactions more limited by mass transport processes than by chemical kinetics itself. Taking crystal growth as an example: microgravity is conducive to growing high-quality crystals, while in the absence of gravity as a ‘natural stirrer’, we need to control the supersaturation more precisely to achieve favorable results.

The sixth challenge I found was that it is very difficult to conduct precise operations in space. Since the CSS operates in a microgravity environment, both people and objects are in a floating state. Routine procedures such as securing objects, transferring liquids, mixing samples, separation, and cleaning all become extremely complex. This demands that astronauts possess strong body posture control, keen observation skills, and precise manual dexterity.

Taking plant cultivation as an example: under weightless conditions, soil particles tend to scatter, and seeds cannot naturally adhere closely to the soil; water–gas regulation is also challenging—water easily accumulates into films that hinder root respiration, yet it can also be excessively absorbed by the substrate, leading to water deficiency in the roots. Thus, we need to tailor precise control methods according to the growth habits of different plants in order to ensure their healthy germination, growth, and harvest in space.

Regardless of all these challenges, with the support of ground-based research teams, our crew proactively overcame various difficulties, and successfully completed all planned scientific research tasks:

1. For the first time in China, we achieved the enclosed in-orbit breeding of four mice (two females and two males), laying a technical foundation for future space mammalian experiments.
2. Through intelligent recognition of facial micro-expressions, we realized non-contact detection of physiological indicators in orbit, and conducted a preliminary exploration of techniques for assessing astronauts’ exercise-induced fatigue.
3. For the first time in China, we achieved in-orbit aeroponic cultivation of wheat and cherry tomatoes, verifying the relevant key technologies.

In addition, the project I helped design—‘*In-Situ* Electrochemical–Optical Study of Lithium-Ion Batteries for Space Applications’—has successfully completed its first phase of research. It is expected to elucidate the mechanisms by which the microgravity environment affects key internal processes of batteries.

Over 210 days of space exploration, Lu Zhang, Fei Wu and I served as both guardians of the CSS and executors of the space laboratory. Every experimental operation, every set of data recorded, and every round of status inspection—that is how we helped to transform the expectations, wisdom, and dedication of the research teams into tangible exploratory practice in the microgravity environment.

It was our great motherland that enabled me to achieve the perfect integration of my scientific ideals and my dream of spaceflight. Now back on the ground, we still feel an overwhelming sense of nostalgia for our work and life aboard the CSS.

I feel deeply honored to have conducted scientific research aboard the Space Station. I would like to express my heartfelt gratitude to our great motherland for its support, to all the personnel across the manned space program, to all the research teams, and to all the friends who have cared for and supported the cause of crewed spaceflight.

The China Manned Space Program has, since its inception, adhered to the principle of ‘building spacecraft to build the space station, and building the station for application’. The emphasis of application lies in producing more and better scientific research outcomes. I hope that an increasing number of researchers will join the cause of China’s manned space program, and fully leverage the invaluable experimental conditions provided by the National Space Laboratory—including microgravity, high radiation, high vacuum, and extreme temperature variations—to contribute our strength to the amazing exploration of science and technology in space!

